# Antimicrobial peptide, cLF36, affects performance and intestinal morphology, microflora, junctional proteins, and immune cells in broilers challenged with *E. coli*

**DOI:** 10.1038/s41598-019-50511-7

**Published:** 2019-10-02

**Authors:** Ali Daneshmand, Hassan Kermanshahi, Mohammad Hadi Sekhavati, Ali Javadmanesh, Monireh Ahmadian

**Affiliations:** 1 Department of Animal Science, Faculty of Agriculture, Ferdowsi University of Mashhad, Mashhad, Iran; 2 School of Environmental and Rural Science, University of New England, Armidale, NSW 2351 Australia; 3 Department of Pathobiology, Ontario Veterinary College, University of Guelph, Guelph, Ontario Canada

**Keywords:** Bacterial infection, Infection

## Abstract

This study investigated the effects of an antimicrobial peptide (AMP), cLF36, on growth performance and the histophysiological changes of the intestine in *E. coli*-challenged broiler chickens. A total number of 360 day old male chicks were randomly assigned to 4 groups of 6 replicates as follows: T1) negative control diet based on corn-soybean meal without *E. coli* challenge and additives; T2) positive control diet based on corn-soybean meal and challenged with *E. coli* without any additives; T3) positive control diet challenged with *E. coli* and supplemented with 20 mg AMP (cLF36)/kg diet; T4) positive control diet challenged with *E. coli* and supplemented with 45 mg antibiotic (bacitracin methylene disalicylate)/kg diet. Results showed that T3 improved growth performance and the jejunal morphology of *E. coli*-challenged chickens similar to those of T4. While antibiotic non-selectively decreased the population of ileal bacteria, AMP increased the population of *Lactobacillus spp*. and decreased harmful bacteria in the ileum of *E. coli*-challenged chickens. Supplementing *E. coli*-challenged chickens with AMP improved the gene expression of immune cells and upregulated the expression of tight junction proteins compared to other challenged groups. In conclusion, although cLF36 beneficially affected growth performance and the intestinal morphology of *E. coli*-challenged chickens similar to those of the antibiotic group, this AMP drastically improved the intestinal microbiome, immune cells, and junctional proteins compared to other *E. coli*-challenged birds, and can be nominated as an alternative for growth promoter antibiotics.

## Introduction

*Escherichia coli* (*E. coli*) is a Gram-negative anaerobic bacterium which may play significant roles as the commensal inhabitant of the gastrointestinal microbiota of poultry^1,2^, while pathogenic strains of *E. coli* can induce intestinal or extra-intestinal diseases^3^. Enteric diseases resulting from the colonization of pathogenic *E. coli* in the gastrointestinal tract of farm animals including poultry causes organ lesion, perihepatitis, airsacculitis, and pericarditis^4^, which lead to growth retardation, mortality and eventually significant economic losses. A common solution to compensate such growth delay is to add antibiotic growth promoters to feed or water of birds, while antibiotic residues in poultry products and the emergence of antibiotic-resistant pathogens have caused consumers concerns^5^. These concerns have resulted in restriction or ban of antibiotic growth promoters in the poultry industry of many countries, especially in Europe^6,7^. Antimicrobial peptides (AMPs) have been recently introduced as potential alternatives to antibiotic growth promoters^8^.

In general, AMPs are small biological molecules (<10 kDa) containing 12–50 amino acids and having broad-spectrum antimicrobial activity against bacteria, some viruses, and fungi^9^. The beneficial effects of AMPs on growth performance, gut morphology, nutrient digestibility, intestinal microflora and immune functions of farm animals have been shown previously^10–12^. More recently, a potent chimeric peptide has been extracted from camel lactoferrin (cLF36) in our lab; its antibacterial^13,14^ and anticancer^15^ characteristics have been demonstrated in previous *in vitro* studies. Although previous research studied the effects of AMPs on different health attributes of animal models in normal conditions, little data is available regarding the effects of AMPs on *E. coli*-challenged animals, to the best of our knowledge. Therefore, the objective of the present study was to evaluate cLF36 as an alternative to growth promoter antibiotics on growth performance and intestinal morphology microflora, immune cells, and barrier proteins in broiler chickens challenged with *E. coli*, as an animal model for infectious disease.

## Results

### Growth performance

The effects of treatments on growth performance attributes are shown in Table 1. Challenging chickens with *E. coli* decreased (P < 0.05) ADG and impaired (P < 0.05) FCR compared to the NC group. Birds receiving antibiotic had the highest (P < 0.05) daily gain at each rearing interval and over the whole period, while AMP-fed birds had similar weight gain to the NC over the whole experimental period. Although antibiotic increased (P < 0.05) ADFI compared to other treatments at first 10 days of age, none of the treatments affected feed intake at the end of the experiment. Supplementing challenged chickens with AMP improved (P < 0.05) FCR compared to the NC group while having similar results as the antibiotic group.

**Table 1:** Effects of treatments on growth performance of broiler chickens from 0 to 24 days of age.

Treatment	ADG^2^ (g)			ADFI (g)			FCR (g/g)		
	0–10	11–24	0–24	0–10	11–24	0–24	0–10	11–24	0–24
NC^1^	16.18^b^	47.60^b^	63.80^b^	22.18^b^	74.70	96.46^ab^	1.37^b^	1.56^b^	1.51^b^
PC	15.08^c^	44.56^c^	59.64^c^	22.42^b^	74.76	94.38^b^	1.48^a^	1.67^a^	1.58^a^
AMP	16.98^ab^	48.16^b^	65.12^b^	22.52^b^	72.12	92.62^b^	1.33^b^	1.50^b^	1.42^c^
Antibiotic	17.32^a^	50.60^a^	67.92^a^	23.86^a^	76.64	100.50^a^	1.38^b^	1.51^b^	1.48^bc^
SEM^3^	0.222	0.541	0.737	0.185	0.632	0.923	0.017	0.019	0.015
P-value	0.001	0.001	0.001	0.005	0.077	0.006	0.007	0.005	0.001

^a,b^Values within a column with different letters differ significantly (P < 0.05).

^1^NC: negative control group received corn-soybean meal diet without any challenge and additives; PC: positive control group received NC diet inoculated with *E. coli* without any additives; AMP: PC received group supplemented with 20 mg antimicrobial peptide/ kg diet; Antibiotic: PC received group supplemented with 45 mg antibiotic (bacitracin methylene disalicylate)/ kg diet.

^2^ADG: average daily gain; ADFI: average daily feed intake; FCR: feed conversion ratio.

^3^SEM: standard error of means (results are given as means of 6 pens of 15 birds/treatment).

### Intestinal morphology

Table 2 summarizes the effects of treatments on villi morphology in the jejunum of chickens. Birds challenged with *E. coli* had lower (P < 0.05) VH, thinner (P < 0.05) VW, and lesser (P < 0.05) VSA compared to the NC birds. At 24 days of age, antibiotic and AMP improved (P < 0.05) VH and VSA compared to control group. Experimental diets had no significant effects on CD and VH/CD at either 10 or 24 days of age.

**Table 2:** Effects of treatments on villi morphology (µm) in the jejunum of broiler chickens at 10 and 24 days of age.

Treatment	Day 10					Day 24				
	VH^2^	VW	CD	VH/CD	VSA (mm)	VH	VW	CD	VH/CD	VSA (mm)
NC^1^	583^a^	161^a^	144	4.31	295.76^a^	1017^b^	174^a^	187	5.68	557.02^b^
PC	455^b^	141^b^	125	3.65	201.10^b^	827^c^	153^b^	201	5.04	396.92^c^
AMP	643^a^	177^a^	138	4.84	356.50^a^	1167^a^	187^a^	171	6.06	671.47^a^
Antibiotic	640^a^	172^a^	121	5.11	326.72^a^	1175^a^	186^a^	180	6.49	688.78^a^
SEM^3^	22.512	3.958	6.602	0.286	16.473	38.576	4.249	10.575	0.314	32.201
P-value	0.004	0.001	0.610	0.306	0.001	0.001	0.001	0.816	0.448	0.001

^a,b^Values within a column with different letters differ significantly (P < 0.05).

^1^NC: negative control group received corn-soybean meal diet without any challenge and additives; PC: positive control group received NC diet inoculated with *E. coli* without any additives; AMP: PC received group supplemented with 20 mg antimicrobial peptide/kg diet; Antibiotic: PC received group supplemented with 45 mg antibiotic (bacitracin methylene disalicylate)/kg diet.

^2^VH: villus height; VW: villus width; CD: crypt depth; VH/CD: the ratio of VH to CD; VSA: villus surface area.

^3^SEM: standard error of means (results are given as means (n = 12) for each treatment).

### Bacterial population

The effects of experimental diets on ileal bacterial populations are shown in Table 3. Challenging chickens with *E. coli* increased (P < 0.05) the population of harmful bacteria (i.e. *E. coli* and *Clostridium spp*.) and decreased (P < 0.05) the colonization of beneficial bacteria (i.e. *Lactobacillus spp*. and *Bifidobacterium spp*.) compared to the NC group. At d 10, antibiotic decreased (P < 0.05) the population of *Lactobacillus spp*. and *Bifidobacterium spp*., while this antibiotic-supplemented diet reduced (P < 0.05) all bacterial populations at d 24 compared to the NC group. Birds supplemented with AMP had the highest (P < 0.05) population of *Lactobacillus spp*. and showed a decrease (P < 0.05) in the ileal colonization of *E. coli* and *Clostridium spp*. at 24 days of age as compared to birds fed the PC diet.

**Table 3:** Effects of treatments on ileal microflora (log_10_ CFU g^−1^) in broilers at 10 and 24 days of age.

Treatments	Day 10				Day 24			
	*E. coli*	*Lactobacillus spp*.	*Bifidobacterium spp*.	*Clostridium spp*.	*E. coli*	*Lactobacillus spp*.	*Bifidobacterium spp*.	*Clostridium spp*.
NC^1^	4.05^b^	6.84^ab^	7.04^a^	1.55^bc^	4.50^b^	7.13^b^	7.45^a^	1.70^b^
PC	5.25^a^	5.71^bc^	5.21^b^	2.17^a^	5.51^a^	6.24^c^	5.96^bc^	2.10^a^
AMP	4.03^b^	7.23^a^	6.35^ab^	1.80^b^	4.13^bc^	8.51^a^	6.90^ab^	1.67^b^
Antibiotic	4.04^b^	5.36^c^	5.36^b^	1.35^c^	3.21^c^	6.21^c^	6.11^c^	1.32^c^
SEM^2^	0.179	0.231	0.256	0.087	0.237	0.187	0.219	0.077
P-value	0.015	0.001	0.015	0.001	0.001	0.004	0.005	0.001

^a–c^Values within a column with different letters differ significantly (P < 0.05).

^1^NC: negative control group received corn-soybean meal diet without any challenge and additives; PC: positive control group received NC diet inoculated with *E. coli* without any additives; AMP: PC received group supplemented with 20 mg antimicrobial peptide/kg diet; Antibiotic: PC received group supplemented with 45 mg antibiotic (bacitracin methylene disalicylate)/kg diet.

^2^SEM: standard error of means (results are given as means (n = 12) for each treatment).

### Gene expression of immune cells and tight junction proteins

**Figure 1:**
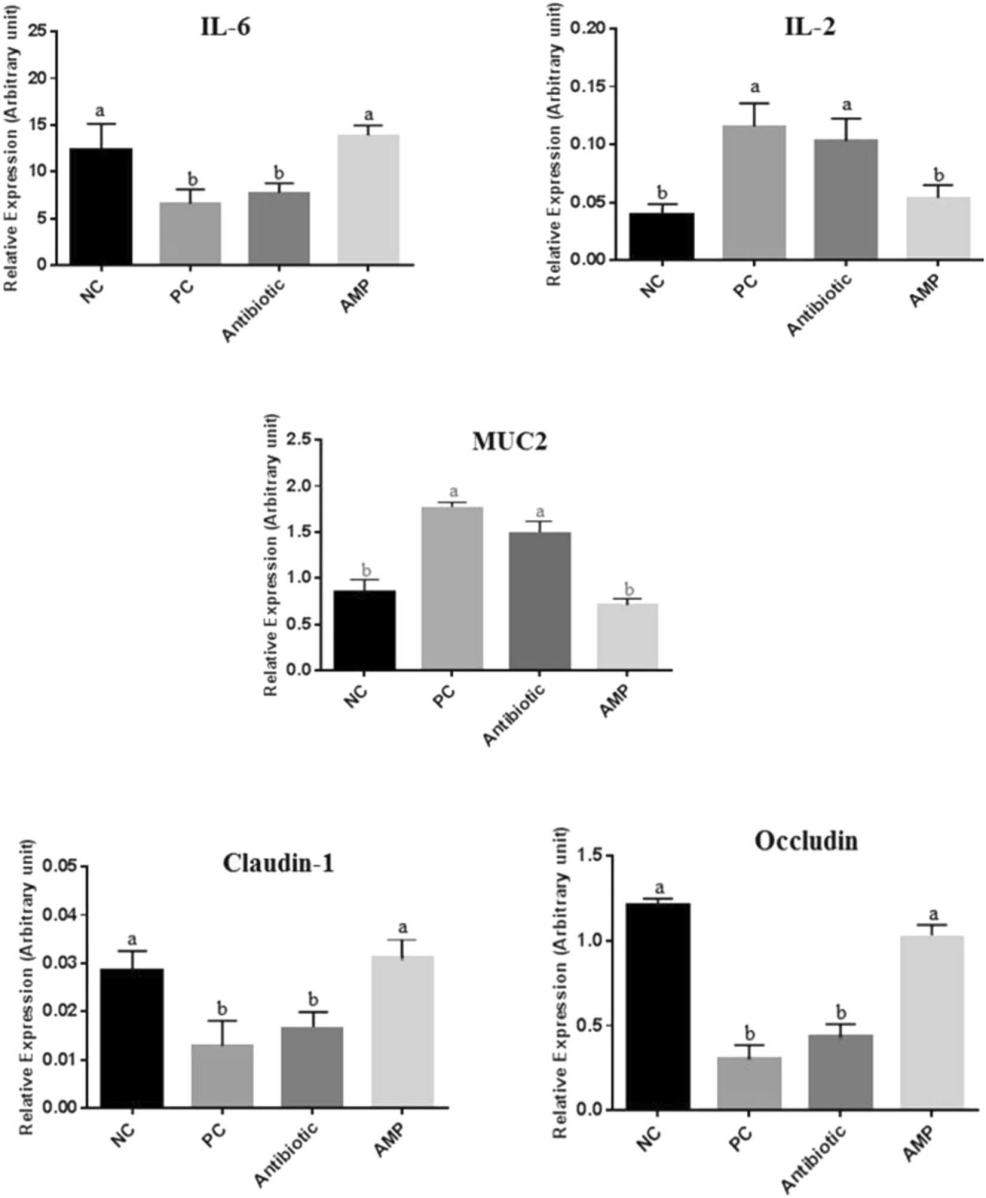
Effects of treatments on the expression of different genes in the jejunum of broiler chickens on day 24. Samples were analyzed using qPCR, and GAPDH and β-actin were used as the reference genes. Abbreviations as follows: IL-6, interleukin 6; IL-2, interleukin 2; MUC2, mucin 2; NC, negative control birds received a corn-soybean meal basal diet without AMPs, antibiotic and *E. coli* challenge; PC, positive control birds received NC diet and orally challenged with one ml of *E. coli* containing 1 × 10^8^ cfu/ml; Antibiotic, birds received PC diet and supplemented with 45 mg antibiotic (bacitracin methylene disalicylate)/kg diet; AMP, birds received PC diet and supplemented with 20 mg peptide/kg diet. The letters on the bar mean show significant difference (P < 0.05).

The effects of experimental diets on gene expression of immune cells and tight junction proteins are shown in Fig. 1. Challenging chickens with *E. coli* increased (P < 0.05) IL-2 and MUC2 expression, but decreased (P < 0.05) IL-6 expression in the jejunum compared to the NC chickens. Adding AMP to the diet resulted in a reduction (P < 0.05) of IL-2 and MUC2 expression and upregulated (P < 0.05) the expression of IL-6 in the jejunum of *E. coli*-challenged chickens. Chickens challenged with *E. coli* had the lowest (P < 0.05) expression pattern of claudin-1 and occludin in the jejunum, while supplementing the diet with antibiotic upregulated (P < 0.05) the expression of tight junction proteins in the jejunum of *E. coli*-challenged birds. Furthermore, adding antibiotic to the diet of *E. coli*-challenged chickens did not affect the regulation of immune cells and tight junction proteins in the intestine.

## Discussion

Increasing concerns of antibiotic resistance have encouraged scientists to search for antibiotic alternatives having the beneficial effects of antibiotics on growth performance and health criteria while preventing transmission of resistance to microbial populations, like those observed in AMPs. The present study was conducted to assess the potency of a new source of peptides to replace antibiotics in the diet of *E. coli*-challenged broiler chickens based on data obtained from productive and health attributes. In agreement with previous studies^16,17^, the current findings showed that challenging chickens with *E. coli* retarded growth and impaired performance, while supplementing the diet with AMP attenuated the negative effects of *E. coli* and improved FCR similar to those of antibiotic-fed birds. The beneficial effects of AMPs on growth performance of broilers under normal^11,18^ and stressful conditions^19^ have been observed previously that is consistent with the results of the present study. The morphological characteristics of villi in the jejunum of birds were investigated to find the possible metabolic and physiological action of AMPs on the growth performance of *E. coli*-challenged birds.

The morphology of villi in the small intestine is known as an indicator of gut health^11^. In addition, the intestinal lumen is the main site of nutrients absorption which directly depends on villus morphology and surface area^20^. Morphological analysis in the present study showed that AMP and antibiotic increased VH and VSA in *E. coli*-challenged birds compared to the control group, which is in agreement with previous studies^11,12^. Consistent with the present results, Liu *et al*.^21^ and Bao *et al*.^18^ reported that supplementing the diet with AMPs extracted from pig intestine and rabbit sacculus rotundus, respectively, improved villus morphology in the duodenum and jejunum of broiler chickens. In general, an increase in VH leads to a greater VSA which increases nutrient absorption from the intestinal lumen^22^ and consequently increases growth performance in birds. In the current study, birds supplemented with peptide and antibiotic had better morphological characteristics compared to the control and *E. coli*-challenged chickens, which resulted in significant improvement in growth performance.

The intestinal microbiome can significantly affect host gut health through various mechanisms such as nutrients absorption, villi morphology, intestinal pH, mucosal immunity, and transporter gene expression^23,24^. In the present study, we examined the effects of treatments on the population of two beneficial (*Lactobacillus spp*. and *Bifidobacterium spp*.) and two pathogenic (*E. coli* and *Clostridium spp*.) bacteria in the ileum of chickens. Antibiotics decreased the population of all bacteria, while AMP significantly improved the community of beneficial bacteria and reduced the colonization of harmful ones in the ileum, which is consistent with previous studies^25,26^. Bacitracin methylene disalicylate exerts its antibacterial activity on the bacterial ribosome subunit resulting in protein synthesis inhibition^27^. This decreases the number of bacteria and microbial damage in the gut, since this antibiotic has a wide range of antibacterial action and does not distinguish between types of bacteria^27,28^ While the definite mechanism by which AMPs can affect the microbial population in the gut has not been found, the suggested mechanism explaining the antimicrobial activity of peptides in controlling the microbial community has been attributed to different surface charges of peptides and pathogens. In detail, AMPs have a net positive charge helping them to electrostatically attach to negatively charged bacterial membranes either to destroy these membranes through physical disruption and/or enzymatic digestion or to pass through the lipid bilayer without exerting any damage. This may interfere with intracellular functions like enzyme activity blockage or inhibiting protein and nucleic acid synthesis^29^. Our previous results showed that the AMP studied in the current experiment can attach to the bacterial membrane through electrostatic interactions and physically disrupt bacterial bilayer membranes^13–15^. Consistent with the previous studies^30–32^, the current results showed that AMP can selectively inhibit the growth of bacteria in the gut which may demonstrate the substantial competitive advantage of cLF36 in comparison to antibiotics.

The invasion of pathogenic bacteria into intestinal epithelial cells and mucosal layer induce the gastrointestinal immune cells to produce cytokines which play different roles in the immune responses to pathogens^33^. IL-6 is a multifunctional cytokine that promotes B cell differentiation and T cell activation^34^. Interestingly, IL-6 can play both pro- (i.e. trans-signaling) and anti- (i.e. classic signaling) inflammatory roles under certain conditions depending on the priority of inflammatory response and the pathophysiological context^35,36^. In agreement with previous findings^29,37^, supplemented AMP upregulated the expression of IL-6 in the jejunum of *E. coli*-challenged chickens in the current study. It has been shown that AMPs can induce the differentiation of bone marrow-derived dendritic cells in the intestine to secrete IL-6 against pathogenic bacteria to protect the intestinal layer from ulceration^38,39^, which may explain the high expression of IL-6 observed in AMP-supplemented group in the current study.

IL-2 is another key cytokine involved in the cellular immune response by T-cell proliferation and the induction of T regulatory responses, and also in the stimulation of B lymphocytes proliferation and immunoglobulin secretion^40^. In the current study, the expression of IL-2 in the jejunum of chickens was upregulated in response to *E. coli* challenge, which is in agreement with previous studies in pig and chicken models^41,42^. Supplementation of AMP to the diet downregulated the expression of IL-2 in the jejunum of *E. coli*-challenged chickens, which may suggest the anti-inflammatory effect of cLF36 in the intestine, which has been reported for other kinds of AMPs^29,37^.

Chickens challenged with *E. coli* in the present study showed an upregulated expression of MUC2, which is in line with previous reports^43,44^. It was shown that the expression of MUC2 increased in the infectious challenge to secrete more mucin from goblet cells into the intestinal lumen to support the protective layer between the invading bacteria and the epithelial cells^45^. Adding AMP to the diet downregulated the expression of MUC2 in the jejunum of challenged chickens, which may be attributed to the significant inhibitory effect of cLF36 on *E. coli* colonization in the intestine (as described above), which is in agreement with previous observation^46^. In the current study, antibiotic did not attenuate the negative effects of *E. coli* on MUC2 expression, which is consistent with previous findings showed that antibiotics may eliminate invading pathogens from the intestinal environment, but be unable to restore the normal circumstances of the intestine after pathogen removal^47^.

It has been well-documented that pathogenic bacteria like *E. coli* attack the intercellular barriers and disrupt tight junction proteins including claudin-1 and occludin through various mechanisms including chemical degradation by bacterial proteases^48,49^ or biochemical alterations of actomyosin ring by phosphorylation^50^ or dephosphorylation^51^. This is consistent with the current observations that *E. coli*-challenged birds showed a drastic decrease in the expression of claudin-1 and occludin in the jejunum. However, AMP upregulated the expression of claudin-1 and occludin in the jejunum of *E. coli*-challenged chickens, which is in agreement with previous studies^52,53^ reporting AMPs to improve the intestinal epithelial integrity and permeability in the context of *E. coli* challenge. Although the exact regulatory mechanism of AMPs on tight junction proteins has not been found yet, two possible theories have been suggested. The first theory implies that AMPs may directly activate regulatory proteins (i.e. Rho family) in the intestine of *E. coli*-challenged mice that increases the expression of junctional proteins and enhances the epithelial barrier function^52,54^. The second theory deals with the antibacterial effects of AMPs on pathogens that decrease the junctional protein disruption and improve the epithelial barrier integrity^55^. Interestingly, antibiotic did not increase the expression of claudin-1 and occludin in the jejunum of *E. coli*-challenged chickens in the current study, while we expected that antibiotic upregulated the junctional proteins due to the antibacterial nature of antibiotics (based on the second above-mentioned theory regarding AMP’s antibacterial effects). In agreement with the present findings, Yi *et al*.^54^ demonstrated that antibiotics did not influence the expression of tight junction proteins after pathogens elimination, maybe due to perturbing the intestinal microbial population. Therefore, the findings of present and previous^52,54^ studies may strengthen the possibility of the first theory attributing the beneficial effects of AMPs on epithelial tight junctions to the expression of regulatory proteins, rather than AMPs’ antimicrobial effects.

In conclusion, the results of the present study suggest that an antimicrobial peptide, cLF36, derived from camel milk can improve growth performance, ameliorate the intestinal morphology changes, and restore gut microbial balance in chickens challenged with *E. coli*. In addition, supplemented cLF36 may enhance the immune response to *E. coli* challenge through regulating the expression of cytokines and mucin. Also, cLF36 can improve the intestinal integrity of *E. coli*-challenged chickens by upregulating the expression of tight junction proteins. Therefore, cLF36 can be introduced as an alternative to growth enhancer antibiotics, based on its beneficial effects observed in the current study, while more research is required to find other contributing aspects of this AMP.

## Methods

All experimental protocols involving animals in the present study were approved by Institutional Animal Care and Use Committee of Ferdowsi University of Mashhad (Protocol number 3/42449) and performed following relevant guidelines and regulations to minimize animal pain, suffering, and distress.

### Birds, treatments, and experimental design

Three hundred and sixty 1-day-old male chicks (Cobb 500) were purchased from a local commercial hatchery, weighed and randomly placed in floor pens (1.1 m × 1.3 m) covered with wood shavings. Birds were assigned to 4 treatments with 6 replicates containing 15 birds in each replicate. Treatments were as follow: (1) negative control (NC) birds received a corn-soybean meal basal diet without AMPs, antibiotic, and *E. coli* challenge; (2) positive control (PC) birds received the NC diet and were orally challenged with one ml of *E. coli* containing 1 × 10^8^ cfu/mL; (3) birds received the PC diet supplemented with 20 mg peptide/kg diet (AMP); (4) birds received PC diet and supplemented with 45 mg antibiotic (bacitracin methylene disalicylate)/kg diet (antibiotic). All diets were in mash form and formulated to meet or exceed the minimum requirements of Cobb 500 (Table 4). Birds had free access to feed and water throughout the experiment and the temperature was set at 32 °C for the first 3 days and then gradually reduced to 21 °C by day 25 which kept constant to the end of the experiment (day 24). The lighting program consisted of 23 L:1D during the first 5 days and then gradually changed to 16 L:8D on day 10 and kept constant to the end of the experiment.

**Table 4:** Composition of experimental diets.

Ingredient (%)^1^	Starter (0–10 days)	Grower (11–24 days)
Corn	56.81	58.16
Soybean meal (44.0%)	36.01	34.85
Soybean oil	3.18	3.35
Dicalcium phosphate	1.79	1.65
Limestone	0.97	0.93
Salt	0.35	0.30
Mineral-vitamin premix^2^	0.50	0.50
DL-Methionine	0.17	0.15
L-Lysine HCl	0.22	0.12
Calculated nutrients		
AME (kcal/kg)	3000	3025
Crude protein (%)	21.0	19.0
Calcium (%)	0.90	0.84
Available phosphorus (%)	0.45	0.42
Sodium (%)	0.16	0.16
Methionine (%)	0.50	0.47
Methionine + cysteine (%)	0.98	0.86
Lysine (%)	1.32	1.18

^1^Antibiotic (45 mg bacitracin methylene disalicylate/kg diet) and peptide (20 mg/kg diet) were added on top and thoroughly mixed.

^2^Added per kg of feed: vitamin A, 7,500 UI; vitamin D3 2100 UI; vitamin E, 280 UI; vitamin K3, 2 mg; thiamine, 2 mg; riboflavin, 6 mg; pyridoxine, 2.5 mg; cyanocobalamin, 0.012 mg, pantothenic acid, 15 mg; niacin, 35 mg; folic acid, 1 mg; biotin, 0.08 mg; iron, 40 mg; zinc, 80 mg; manganese, 80 mg; copper, 10 mg; iodine, 0.7 mg; selenium, 0.3 mg.

### AMP production

The AMP used in the present study was derived from camel lactoferrin (cLF) consisting of 42 amino acids which was generated in our lab recently (for more details regarding the peptide cLF chimera production, review previous works^13–15^). Briefly, preparation of recombinant plasmid vector was conducted through transforming synthetic cLFchimera into DH5α bacterium^13–15^. Next, the latter bacterial colonies were cultured to harvest plasmid extraction. Then, the recombinant vector was transferred into *E. coli* (DE3) as an expression host and cultured in 2 mL Luria-Bertani broth (LB) medium for overnight according to standard protocol^56^. In the next step, cultured materials were inoculated in 50 mL LB and incubated at 37 °C with shaking at 200 rpm. Then, isopropyl-β-D-thiogalactopyranoside (IPTG) was added to a final concentration of 1 mM and incubated at 37 °C for 6 h after IPTG induction. Periplasmic protein was collected at different times after IPTG induction (2, 4 and 6 h) according to the method described by de Souza Cândido *et al*.^57^ and analyzed on 12% SDS-PAGE. To purify expressed peptide, Ni-NTA agarose column was used based on the manufacturer’s instruction (Thermo, USA). The quality of purified recombinant peptide was assessed on a 12% SDS-PAGE gel electrophoresis, while the Bradford method^58^ was used to analyze the quantity of recombinant peptide. More recently, an *E. coli* expression system^14^ was developed in our laboratory that is able to produce 0.42 g/L of recombinant peptide. In the current study, 4 g peptide previously obtained from the recombinant *E. coli* were purified, lyophilized, and thoroughly mixed with 1 kg soybean meal and then supplemented to the relevant experimental diets. The inhibitory effects of this AMP on various plant^13^ and poultry^14^ pathogens were recently observed in *in vitro*.

### *E*. *coli* challenge

The method of *E. coli* challenge was explained in details elsewhere^17^ with some minor differences. In summary, a suspension of *E. coli* (ATCC 31616) was cultured on MacConkey agar plates (Merck, Germany) for 24 h at 37 °C, and pink, round medium-sized colonies were picked as *E. coli* suspect colonies to prepare the inocula. Next, *E.coli* K99 was inoculated in LB medium and incubated at 37 °C for 24 h. Cell bacteria density was determined in the medium by the subculture of bacteria after making a serial dilution. Bacteria were adjusted to 10^8^ cfu/ml by diluting in 0.5% peptone solution. On d 7, chicks were orally challenged with 1 ml of prepared inoculation containing 1 × 10^8^ cfu *E. coli*, while non-challenged chicks received 1 ml of sterile peptone water.

### Growth performance

Body weight (BW) and feed remaining of each pen were weighed on days 10 and 24 to measure the average daily gain (ADG), average daily feed intake (ADFI) and feed conversion ratio (FCR) over the specific and entire periods of experiment (0–10, 11–24, and 0–24 days of age). Mortality per pen was recorded daily in order to adjust FCR accordingly.

### Sample collection

Two birds from each pen (12 birds/treatment) were randomly selected on days 10 and 24, euthanized by cervical dislocation, the viscera was excised, the intestine was discreetly separated from the whole viscera, and the adherent materials were precisely removed. The ileum was gently pressed to aseptically collect ileal content into sterile tubes for microbiological analysis. A section (about 5 cm) from mid-jejunal tissues was meticulously separated for morphological analysis. A 2 cm section from the mid-jejunum was detached, rinsed in cold phosphate-buffered saline (PBS), immediately immersed in RNAlater (Qiagen, Germantown, MD) and stored at −20 °C for subsequent gene expression determination.

### Intestinal morphology

The method described by Daneshmand *et al*.^59^ was used to prepare samples for morphometry analysis. In summary, jejunal and ileal samples were stored in a 10% formaldehyde phosphate buffer for 48 h. Then, the samples were trimmed and processed on a tissue processor (Excelsior™ AS, Thermo Fisher Scientific, Loughborough, UK), fixed in paraffin using an embedder (Thermo Fisher Histo Star Embedder, Loughborough, UK) and cut with a microtome (Leica HI1210, Leica Microsystems Ltd., Wetzlar, Germany) to a slice of 3 μm, placed on a slide and dehydrated on a hotplate (Leica ASP300S, Leica Microsystems Ltd., Wetzlar, Germany). Then, the prepared samples were dyed with hematoxylin and eosin and examined under a microscope (Olympus BX41, Olympus Corporation, Tokyo, Japan). A total of 8 slides were prepared from the jejunal segment per bird, and 10 individual well-oriented villi were measured per prepared slide (80 villi/bird). The average of slide measurements per sample was stated as a mean for each bird. Villus width (VW) was measured at the base of each villus; villus height (VH) from the top of the villus to the villus-crypt junction, crypt depth (CD) from the base of the adjacent villus to the sub-mucosa, the ratio of VH to CD and villus surface area were calculated.

### Microbial count

The method explained by Kermanshahi *et al*.^60^ was used to count the populations of *E. coli*, *Clostridium spp*., *Lactobacillus spp*., and *Bifidobacterium spp*. in the collected ileal content. Briefly, the ileal contents of a sample were thoroughly mixed, serially diluted 10-fold from 10^−1^ to 10^−7^ with sterile PBS and homogenized for 3 minutes. Then, dilutions were plated on different agar mediums. Regarding the enumeration of bacteria, *Lactobacillus spp*. and *Clostridium spp*. dilutions were plated on MRS agar (Difco, Laboratories, Detroit, MI) and SPS agar (Sigma, Germany) and anaerobically cultured at 37 °C for 48 h. Black colonies in SPS agar medium were recorded as the count of *Clostridium spp*. Eosin Methylene Blue (EMB) agar (Merck, Darmstadt, Germany) and BSM agar (Sigma-Aldrich, Germany) were used to cultivate *E. coli* and *Bifidobacterium spp*. respectively, and incubated at 37 °C for 24 h. All microbiological analyses were performed in triplicate, average values were used for statistical analyses and results were expressed in colony-forming units (Log_10_ cfu/g of ileal content).

### RNA extraction and gene expression

The procedure of RNA extraction and gene expression was described previously^61^. In summary, total RNA was extracted from chicken jejunum sampled on day 24 using the total RNA extraction kit (Pars Tous, Iran) following the manufacturer’s instructions. Purity and quality of extracted RNA were evaluated using an Epoch microplate spectrophotometer (BioTek, USA) based on 260/230 and 260/280 wavelength ratios, respectively. Genomic DNA was removed using DNase I (Thermo Fisher Scientific, Austin, TX, USA). The complementary DNA (cDNA) was synthetized from 1 µg of total RNA using the Easy cDNA synthesis kit (Pars Tous, Iran) following the manufacturer’s protocol.

Gene expression of two references (GAPDH and β-actin) and five targets (Interleukin-1 [IL-1], IL-6, mucin2 [MUC2], Claudin-1 [CLDN1], and Occludin [OCLN]) genes were determined by quantitative real-time PCR (qRT-PCR) based on MIQE guidelines^62^. Each reaction was performed in a total volume of 20 μl in duplicate using an ABI 7300 system (Applied Biosystems, Foster City, CA) and 2 × SYBR Green Real Time-PCR master mix (Pars Tous, Iran). Primer details are shown in Table 5. All primers were designed according to MIQE criteria^62^ regarding amplification length and intron spanning. All efficiencies were between 90 and 110% and calculated R2 was 0.99 for all reactions. The method 2^−ΔΔCt^ Ct^63^ was used to calculate relative gene expression in relation to the reference genes (GAPDH and β-actin).

**Table 5:** Sequences of primer pairs used for amplification of the target and reference genes^1^.

Gene^2^	Strand	Sequence (5′ → 3′)	Ta	Product size (bp)	GenBank Accession No.
IL-2	Forward	TTATGGAGCATCTCTATCATCAGCA	63	122	XM_01576098.1
	Reverse	CCTGGGTCTCAGTTGGTGTGTAG			
IL-6	Forward	CTGTTCGCCTTTCAGACCTACC	63	141	NM_204628.1
	Reverse	GACCACTTCATCGGGATTTATCA			
MUC2	Forward	ATGCGATGTTAACACAGGACTC	60	110	BX930545
	Reverse	GTGGAGCACAGCAGACTTTG			
CLDN1	Forward	CATACTCCTGGGTCTGGTTGGT	60	100	NM_001013611.2
	Reverse	GACAGCCATCCGCATCTTCT			
OCLDN	Forward	CGCAGTCCAGCGGTTACTA	58	178	NM_205128.1
	Reverse	AGGATGACGATGAGGAACCCA			
GAPDH	Forward	TTGTCTCCTGTGACTTCAATGGTG	63	128	NM_204305
	Reverse	ACGGTTGCTGTATCCAAACTCAT			
β-Actin	Forward	CCTGGCACCTAGCACAATGAA	63	175	NM_205518.1
	Reverse	GGTTTAGAAGCATTTGCGGTG			

^1^For each gene the primer sequence for forward and reverse (5′ → 3′), the product size (bp), and the annealing temperature (Ta) in °C are shown.

^2^IL-, interleukin-; MUC2, mucin2; CLDN1, claudin1, OCLDN, occludin; GAPDH, Glyceraldehyde 3-phosphate dehydrogenase.

### Statistical analysis

Data were statistically analyzed in a completely randomized design by ANOVA using the General Linear Model (GLM) procedure of SAS (SAS Inst., Inc., Cary, NC). Tukey’s test was used to compare differences among means of treatments and P values < 0.05 were considered to be significant.

## Data Availability

All data generated or analysed during this study are included in this published article.
